# Molecular basis of apoptotic DNA fragmentation by DFF40

**DOI:** 10.1038/s41419-022-04662-7

**Published:** 2022-03-02

**Authors:** Hyun Ji Ha, Hyun Ho Park

**Affiliations:** 1 College of Pharmacy, Chung-Ang University, Seoul, 06974 Republic of Korea; 2 Department of Global Innovative Drugs, Graduate School of Chung-Ang University, Seoul, 06974 Republic of Korea

**Keywords:** Proteins, Enzymes, X-ray crystallography

## Abstract

Although the functions of CIDE domain-containing proteins, including DFF40, DFF45, CIDE-A, CIDE-B, and FSP27, in apoptotic DNA fragmentation and lipid homeostasis have been studied extensively in mammals, the functions of four CIDE domain-containing proteins identified in the fly, namely DREP1, 2, 3, and 4, have not been explored much. Recent structural study of DREP4, a fly orthologue of mammalian DFF40 (an endonuclease involved in apoptotic DNA fragmentation), showed that the CIDE domain of DREP4 (and DFF40) forms filament-like assembly, which is critical for the corresponding function. The current study aimed to investigate the mechanism of filament formation of DREP4 CIDE and to characterize the same. DREP4 CIDE was shown to specifically bind to histones H1 and H2, an event important for the nuclease activity of DREP4. Based on the current experimental results, we proposed the mechanism underlying the process of apoptotic DNA fragmentation.

## Introduction

Apoptotic DNA fragmentation, resulting in approximately 180-bp fragments, is a biochemical hallmark of apoptotic cell [1]. This process occurs in at least two distinct stages 1) initial large-scale DNA cleavage and 2) the second small-scale DNA fragmentation [2, 3]. Among the several identified endonucleases, apoptosis-inducing factor (AIF) and EndoG are responsible players for initial large scale DNA cleavage [4–6], while DNA fragmentation factor 40 (DFF40) is considered as the main player in the later process of small scale DNA fragmentation during apoptosis [7–9]. In the absence of apoptotic stimuli, the nuclease activity of DFF40 is inhibited by DNA fragmentation factor 45 (DFF45) via the formation of a heterodimer [9, 10]. Once apoptosis is triggered, effector caspases, such as caspase-3, are activated and cleave DFF45, allowing DFF40 to be dissociated from DFF45, enter into the nucleus, and degrade chromosomal DNA [9, 11, 12].

In the complex formation between DFF40 and DFF45, as a part of the inhibition process of DFF45, the cell death-inducing DFF45-like effector domain (CIDE domain), which is the protein interaction module present in both DFF40 and DFF45, is responsible for tight protein-protein interaction [8, 13]. Besides DFF40 and DFF45, three more CIDE domain-containing proteins, including CIDE-A, CIDE-B, and CIDE-3 (FSP27 in mouse), have been identified in mammals, based on sequence homology analysis [14, 15]. CIDE domain-containing proteins mainly function in apoptotic DNA fragmentation; however, various studies have shown them to be involved in lipid metabolism as well [16–19]. The most recent study of CIDE-3 showed that this CIDE domain-containing protein regulated the lipid droplet size and stimulated intracellular lipid deposition [20]. The mechanism of lipidic roles of CIDE domain-containing proteins are totally unknown.

Apoptotic DNA fragmentation is conserved in fly, and DREP4 and DREP1 have been identified as DFF40 and DFF45 homologues, respectively, in fly [21, 22]. Although the functions of DREP2 and DREP3 are not yet clear, DREP2 has been suggested to be involved in memory and learning by localizing in brain synapse [23]. Filament formation of DREP4 and DFF40 via CIDE domain is a critical step in the proper apoptotic DNA fragmentation process [24]. To reveal the mechanism and implication of filament formation in DREP4 and DFF40, the current study aimed to characterize the filament formation of DREP4 CIDE using mutagenesis and electron microscopy. DREP4 CIDE was found to specifically bind to histones H1 and H2, which are important for the nuclease activity of DREP4. Based on our findings, we proposed the complete mechanism of the apoptotic DNA fragmentation process in this report.

## Materials and Methods

### Homology modeling of CIDE domain structures

The methods used for homology modeling of DREP1 CIDE and DREP3 CIDE have been reported previously [25, 26]. Briefly, the CIDE-B structure (PDB ID: 1D4B) was used for modeling DREP1 CIDE and DREP3 CIDE. Electrostatic surfaces and ribbon diagrams were generated using the PyMOL program (The PyMOL Molecular Graphics System, DeLano Scientific, San Carlos, USA).

### Protein expression and purification

The expression and purification of DREP1 (amino acid residues, 10–90), DREP3 (amino acid residues, 111–195), and DREP4 (amino acid residues, 39–130) were performed as described in previous studies [24, 26, 27]. Briefly, each plasmid containing the CIDE domain was transformed into BL21 (DE3) *Escherichia coli* competent cells (Sigma-Aldrich, St. Louis, Missouri, USA), and then expression was induced by treating the bacteria with 0.5 mM isopropyl β-D-thiogalactopyranoside overnight at 20 °C. The bacteria were then collected, resuspended, and lysed by sonication in 50 mL lysis buffer (20 mM Tris-hydrochloride [HCl] pH 7.9, 500 mM sodium chloride [NaCl], and 10 mM imidazole). Next, the bacterial lysate was centrifuged, the supernatant collected, and eventually applied to a gravity-flow column packed with Ni-NTA affinity resin (Qiagen, Hilden, Germany). Unbound bacterial proteins were removed from the column by washing with buffer (20 mM Tris-HCl pH 7.9, 500 mM NaCl, 60 mM imidazole, and 10% glycerol), and the target proteins were eluted using an elution buffer (20 mM Tris-HCl pH 7.9, 500 mM NaCl, and 250 mM imidazole). The protein was further purified using a Superdex 200 gel-filtration column (GE Healthcare, Chicago, Illinois, USA). All the mutant proteins were expressed and purified by the same method as used for the wild-type CIDE domain.

### Oligomerization assay using size-exclusion chromatography

For size-exclusion chromatography, to detect oligomer formation, target protein was applied to a gel-filtration column (Superdex 200 HR 10/30, GE Healthcare) that had been pre-equilibrated with 20 mM Tris-HCl pH 8.0 and 500 mM NaCl. The peak fractions were then collected and subjected to SDS-PAGE. For the salt concentration-dependent oligomerization test, indicated concentrations of salt were added to the buffer for size-exclusion chromatography.

### Multi-angle light scattering (MALS)

The molar mass of DREP4 CIDE domains, including mutants, was determined by MALS. The target protein was injected into a Superdex 200 HR 10/30 gel-filtration column (GE Healthcare) that had been pre-equilibrated with buffer containing different concentrations of NaCl. The chromatography system was coupled to a three-angle light scattering detector (mini-DAWN EOS) and a refractive index detector (Optilab DSP) (Wyatt Technology, Santa Barbara, USA). Data were collected every 0.5 s at a flow rate of 0.2 mL/min and analyzed using the ASTRA program, which gave the molar mass and mass distribution (polydispersity) of the sample.

### Electron microscopy

DREP4 CIDE samples in 50 mM NaCl or 2 M NaCl, after purification by affinity chromatography, were diluted to 0.5 mg/mL. For negative staining, 10 μL of each protein sample was placed on a glow-discharged copper grid and stained with 1% uranyl formate at pH 4.5 for 30 s, and air-dried. The grids were imaged using the Tecnai G² Spirit BioTWIN Transmission Electron Microscope and recorded with an AMT 2k CCD camera (Thermo Fisher Scientific, Sunnyvale, CA, USA).

### Mutagenesis

Site-directed mutagenesis was conducted using a quick-change kit (Stratagene) according to the manufacturer’s protocols. Mutagenesis was confirmed by sequencing. Mutant proteins were prepared using the same method as described above.

### Sequence alignment

The amino acid sequences of CIDE domain were analyzed using Clustal Omega (http://www.ebi.ac.uk/Tools/msa/clustalo/).

### Native-PAGE shift assay

Changes in oligomerization in response to mutations and complex formation were monitored by native (non-denaturing) PAGE on a PhastSystem system (GE Healthcare) with pre-made 8–25% acrylamide gradient gels (GE Healthcare). Separately purified proteins were pre-incubated at 20 °C for 1 h before loading the gel. Coomassie Brilliant Blue was used for staining and detection of the shifted bands.

### DNA binding assay

Purified DREP4 CIDE protein (10 μg) was pre-incubated with 10 μg of linearized plasmid DNA at 25 °C for 30 min in a final volume of 20 μL buffer containing 20 mM Tris-HCl pH 8.0 and 150 mM NaCl. The reaction mix was then run on 1.5% agarose gel for 30 min at 150 V.

### His-tag pull-down assay

Purified DREP4 CIDE and each histone protein were mixed and incubated with Ni-NTA resin for 1 h at 20 °C. After washing the bead with 500 mL washing buffer (20 mM Tris-HCl pH 7.9, 500 mM NaCl, 60 mM imidazole, and 10% glycerol), the bound protein was eluted from the Ni-NTA column and loaded onto SDS-PAGE gels.

## Results and Discussion

### Filament formation of DREP4 CIDE domain is more sensitive to salt concentration than that of other CIDE domains

**Fig. 1:**
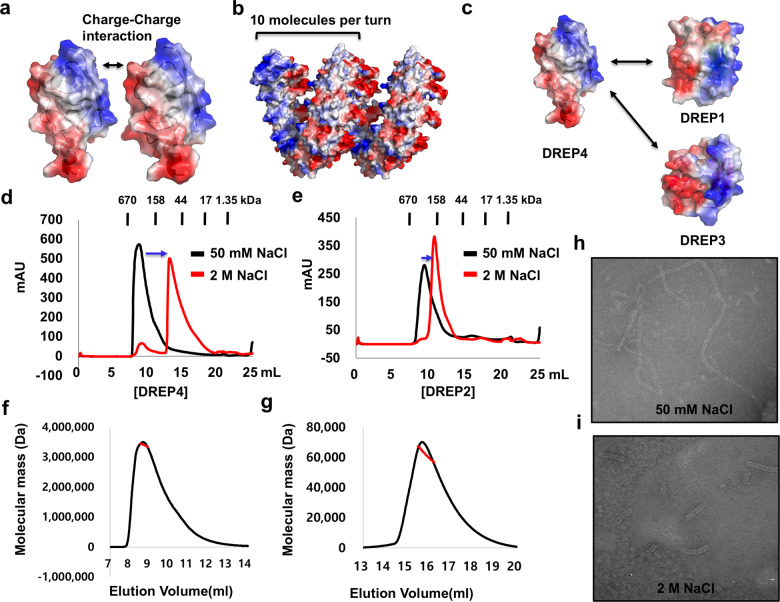
Dynamic property of the helical filament-like structure of DREP4 CIDE. **a** A strategy of homotypic interaction of DREP4 CIDE. The surface electrostatic distribution of DREP4 CIDE is presented. **b** Helical oligomer of DREP4 CIDE. Surface electrostatic distribution of helical oligomer of DREP4 CIDE is presented; 10 molecules of DREP4 CIDE are needed for one turn of helical structure. **c** Heterotypic interaction of DREP4 CIDE. Both DREP1 CIDE and DREP3 CIDE interact with the basic surface of DREP4 CIDE. **d**, **e** Size-exclusion chromatograms of DREP4 CIDE (**d**) and DREP2 CIDE (**e**) in low salt (50 mM NaCl) and high salt (2 M NaCl) conditions. Movement of peaks due to the reduced size of filament particles is indicated by blue arrows. **f**, **g** Multiangle light scattering (MALS) of DREP4 CIDE in low salt (50 mM NaCl) (**f**) and high salt (2 M NaCl) concentrations **g** showing the absolute molecular mass of the protein. Red line indicates experimental absolute molecular mass. (**h**, **i**) Electron microscopy images of negatively stained DREP4 CIDE in low salt (50 mM NaCl) (**h**) and high salt (2 M NaCl) (**i**) conditions.

To fully understand the filament formation process of DREP4 (DFF40 homologue in fly) during apoptotic DNA fragmentation, we characterized DREP4 CIDE domain and its filament states using the currently available helical filament structure of DREP4 CIDE domain [24]. The presence of two oppositely charged surfaces, acidic and basic, is a well-known feature of CIDE domains, including DREP4 CIDE (Fig. 1a) [25, 28]. The filament-like structure of DREP4 CIDE formed by charge-charge interaction among the molecules, and 10 such molecules formed a turn in the helical filament structure (Fig. 1b) [24, 28]. Previous studies have shown both DREP1 and DREP3 to bind to DREP4 CIDE (Fig. 1c) [26]. DREP1 is an inhibitor of DREP4, and the effect of DREP3 on DREP4 is not yet known. During the study of various CIDE domains, we realized that the self-assembly of CIDE domain, especially of DREP4 CIDE, is affected by salt concentration. This feature was quantitatively analyzed by MALS and electron microscopy (EM). As judged by size-exclusion chromatography, DREP4 CIDE formed a highly oligomeric complex in the presence of low salt concentration (50 mM NaCl), and the complex was dissociated at the high salt concentration (2 M NaCl) (Fig. 1d). Although subunit interactions in the helical filament assembly of CIDE domain occurs mainly via charge-charge interaction, which can be disrupted by high concentrations of salt, the sensitivity was different for each CIDE domain. In case of DREP2 CIDE, dissociation of filament structure at high salt concentration was not dramatic, compared to that in DREP4 CIDE, indicating that self-assembly of DREP4 CIDE is more sensitive to salt concentration (Fig. 1d, e).

To quantitatively analyze the salt-dependency of the self-assembly of DREP4 CIDE, we adopted MALS, which can determine the absolute molecular mass of the particle. The molecular masses of DREP4 CIDE in 50 mM NaCl and 2 M NaCl were calculated using MALS with the basal information that the theoretical molecular weight of DREP4 CIDE is 11.32 kDa. MALS indicated that the complex masses in 50 mM NaCl and 2 M NaCl were 334.4 kDa (6.40% fitting error) and 61.5 kDa (1.35% fitting error), respectively, thereby indicating that self-assembly of DREP4 CIDE is sensitive to and dependent on salt concentration (Fig. 1f, g). The sensitivity of self-assembly of DREP4 CIDE was further confirmed by EM of the negatively stained samples. The experiment showed that DREP4 CIDE forms long filament-like particles in 50 mM NaCl (Fig. 1h) and much smaller filaments in 2 M NaCl (Fig. 1i), thereby validating the dependence of self-polymerization of DREP4 CIDE on salt concentration.

### Hinge loop and linker helix are located in between CIDE domain and nuclease domain of apoptotic nucleases

**Fig. 2:**
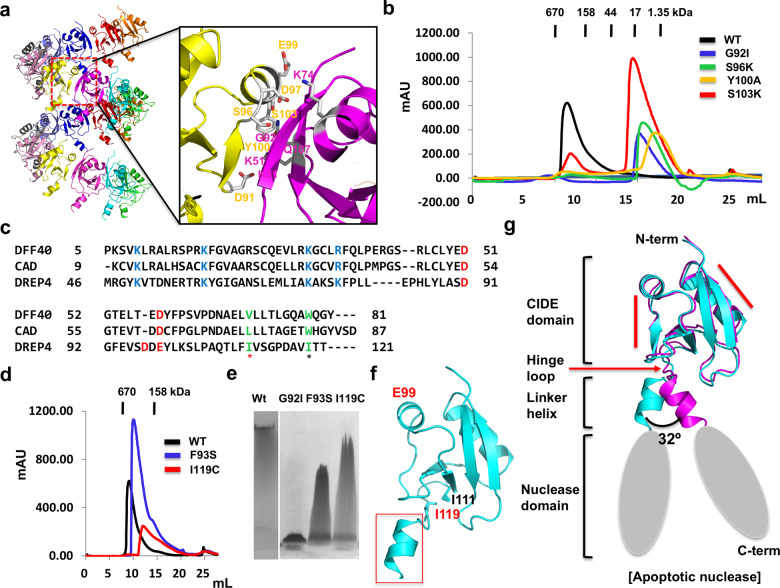
Mutational analysis of homo-oligomeric interface and monomeric form of DREP4 CIDE. **a** Analysis of homo-oligomeric interface of DREP4 CIDE. Close-up view of the interacting residues in the interface between two DREP4 CIDE is shown in the right panel. Residues involved in the contact are labeled. **b** Size-exclusion chromatogram of DREP4 CIDE and its various mutants (mutated in the residues located in the interface). **c** Sequence alignment of DREP4 CIDE homologues. Residues conserved and involved in the formation of helical oligomer are colored blue for basic and red for acidic residues. W78 in DFF40 and aligned sequences (I119 on DREP4) are indicated by black star. I111 in DREP4 and aligned sequences are indicated by red star. **d** Size-exclusion chromatogram of DREP4 CIDE and its mutants, F93S (corresponding to T53 on DFF40) and I119C (corresponding to W78 on DFF40). W78C mutants diminished the activity of DFF40, whereas T53L mutant did not have any effect. **e** Native-PAGE of DREP4 CIDE and its mutants G92I, F93S, and I119C. **f** Position of two residues, whose mutations diminish the enzymatic activity but still allow the formation of a helical oligomer. **g** Existence of hinge loop and linker helix in apoptotic nuclease. Red lines indicate other CIDE-binding regions for the formation of helical oligomer. The structure of two DREP4 CIDEs in the asymmetric unit were superposed to compare the structure.

Previously, we had verified the helical filament assembly of DREP4 CIDE by mutagenesis of charged residues, thereby disrupting the charge-charge interaction between subunits [24]. To confirm the interaction interface in the filament-like structure, here we mutated the non-charged residues (G92I, S96K, Y100A, and S103K) in the interface of the subunits (Fig. 2a). All interface-disrupted mutants eluted at approximately 16 mL in size-exclusion chromatography, indicating that none of them formed a proper filament complex (Fig. 2b). Interestingly, the G55I mutant of CAD (G52I mutant of DFF40), which corresponds to G92I in DREP4 (Fig. 2c), diminished the activity of CAD/DFF40 as shown in a previous study [29], indicating that the filament structure of apoptotic nuclease is critical for its DNA cleavage activity.

Besides the charged residues (K9A, D51A, E57K, and D58K) and G52 residue in the interface, W78C mutant in DFF40, which is not located in the interface, had also been reported to diminish the nuclease activity [29, 30]. To analyze the reason behind W78 being critical for the activity of DFF40, we found the corresponding residue of W78 in DREP4 and found I119 in DREP4 to be the one that aligned with W78 in DFF40 by sequence and structure alignment (Fig. 2c). The T53L mutant in DFF40 (corresponding residue of F93S in DREP4), which is not located in the interface and has no effect on the activity of DFF40 [29], was used for further analysis. Both F93S and I119C mutants of DREP4 CIDE formed oligomeric complex, as seen by size-exclusion chromatography, although the tailing of the main peak was detected, unlike in wild-type DREP4 CIDE (Fig. 2d). The disruption effect of each mutant on self-oligomerization was confirmed by native-PAGE. The latter indicated that while F93S and I119C produced the homo-oligomeric complex band, G92I, used for the control experiment, failed to produce a highly oligomerized band (Fig. 2e, Supplementary Fig. 1). Smear tailing bands in the F93S and I119C loading lanes, which might be produced due to the heterogeneous population of oligomeric form, were seen in native-PAGE (Fig. 2e and Supplementary Fig. 1). To answer the importance of W78 residue on DFF40, which is corresponded to I119 on DREP4, for the activity of DFF40 and DREP4, we mapped I119 on the structure of DREP4 CIDE. As indicated, it was found to be located on the loop at the end of CIDE domain and formed hydrophobic interaction with the neighboring I111 residue (Fig. 2f). Since the structure of DREP4 CIDE contains an extra helix, unlike that of other CIDE domains, positional variation of the extra helix was analyzed by superposition of all 10 subunits detected in the filament-like structure of DREP4 CIDE. Analysis showed that the biggest locational difference between two extra helices from different subunits was a rotation by 32°, which indicated that the extra helix could be moved in different directions (Fig. 2g). If the nuclease domain of an apoptotic nuclease is linked to the end of the extra helix, the location of the nuclease domain can be variable (Fig. 2g). Based on this structural speculation, we called the extra helix a linker helix and the loop in between the CIDE domain and the linker helix a hinge loop. Therefore, N-terminal CIDE domain was shown to be linked to C-terminal nuclease domain via a hinge loop and a linker helix, which can move freely (Fig. 2g). The movement of hinge loop and linker helix might be critical for binding to the cutting site of DNA due to adjustment of the position of the nuclease domain.

### Basic surface of DREP4 binds to acidic surface of DREP1, and the interaction is not disrupted by linker loop defect

**Fig. 3:**
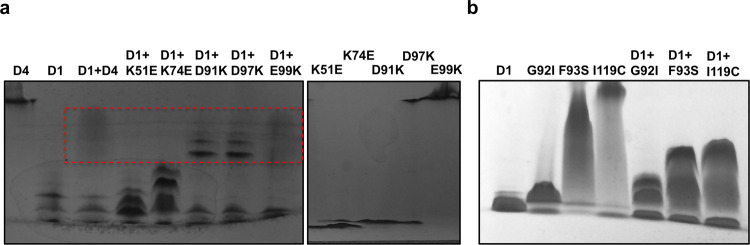
Effect of DREP4 mutation on its binding with DREP1. Native-PAGE showing the interaction of DREP1 with interface residue mutants (**a**) and other mutants (**b**) of DREP4. The loaded protein samples are indicated above the corresponding lanes. The bands formed due to complex formation are indicated by red-dashed box.

DREP1 is known as DFF45 homologue in fly [31]. The interaction of DREP4 with DREP1 via CIDE domain and the position of their heterodimeric interaction had been determined previously in vitro [22, 32]. To understand the relationships between helical filament assembly of DREP4 and DREP1 interaction, and linker loop defect in DREP4 and DREP1 interaction, we performed native-PAGE and explored the complex formation. As shown in Fig. 3a, basic surface mutants of DREP4, including K51E and K74E, did not form a complex with DREP1 CIDE, whereas acidic surface mutants, including D91K, D97K, and E99K, did, indicating that the basic surface of DREP4 interacts with the acidic surface of DREP1. We also analyzed the interaction of G92I, F93S, and I119C with DREP1 CIDE. Since G92 is located on the basic surface of DREP4, G92I mutant failed to form a helical filament structure and to interact with DREP1 CIDE, whereas F93S and I119C of DREP4 did not affect the filament formation of DREP4 nor its interaction with DREP1 CIDE (Fig. 3b). All uncropped gel figures for generating Fig. 3 was provided at Supplementary Fig. 2. Complex formation of various DREP4 mutants with DREP1 CIDE, analyzed by native-PAGE, was further confirmed by size-exclusion chromatography followed by SDS-PAGE. Based on the location of the eluted peak and the pattern of co-migrated bands in SDS-PAGE, we concluded that K51E, K74E, and G92I failed to form a complex with DREP1 CIDE, whereas D91K, D97K, E99K, F93S, and I119C still did (Supplementary Fig. 3a–h). The results indicated that only basic surface of DREP4 CIDE was critical for binding to DREP1 CIDE and the linker loop was not so important in this regard.

### Basic building unit of the filament structure of CIDE domain is a dimer

**Fig. 4:**
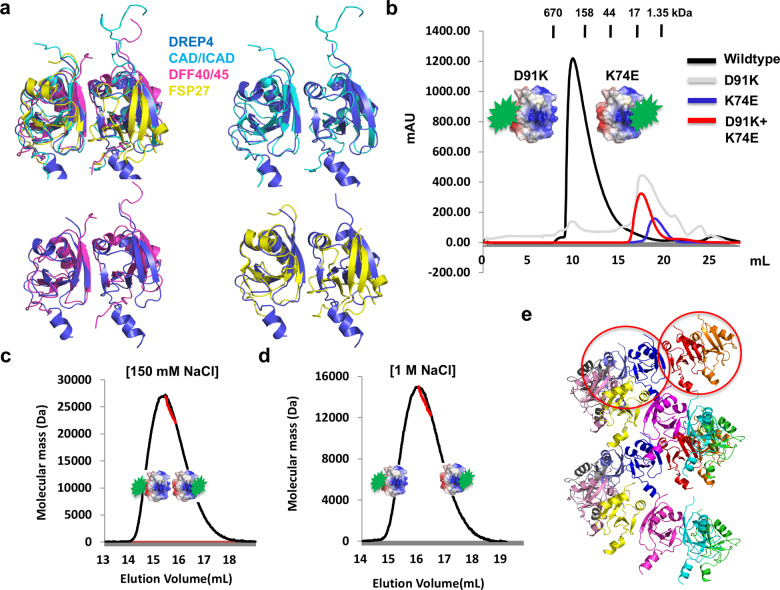
Homo-dimeric DREP4 CIDE as a basic building block of helical oligomer. **a** Previously elucidated homo- or heterodimeric structure of CIDE domain. Structure alignment of homodimeric form of DREP4 CIDE with the DFF40-DFF45 heterodimer, the CAD-ICAD heterodimer, and the FSP27 homodimer. **b** Size-exclusion chromatogram of the wild-type DREP4 CIDE, mutants, and two-mutant mixture. Acidic and basic patch-disrupted mutants are introduced. **c** and **d** Multiangle light scattering (MALS) measurement of the mixture of two mutants, K74E and D91K, in low salt (150 mM NaCl) (**c**) and high salt (1 M NaCl) (**d**) conditions. Redline indicates experimental absolute molecular mass. Expected association in low salt condition (**c**) and dissociation in high salt condition (d) are shown by surface figures. **e** Cartoon figure of helical oligomer of DREP4 CIDE. Homodimer, which is the tentative basic building unit of oligomeric filament-like structure, is indicated by red circle.

Before the helical filament structure was introduced by structural studies of DREP2 and DREP4 [2, 24], dimeric form was suggested to be the major hetero- or homo-oligomeric form of CIDE domain, based on the heterodimeric complex structures between DFF40 CIDE and DFF45 CIDE [33], and between CAD and ICAD [34], as well as the homo-dimeric structure of the FSP27 CIDE domain [35]. The interaction strategy of the dimers was the same as that of hired by filament structure of DREP4 CIDE. One of the dimers in the filament structure of DREP4 CIDE is almost identical to the hetero- or homodimer structures of other CIDE domains, exhibiting a root mean square deviation (R.M.S.D.) of 2.2 Å with CAD/ICAD heterodimer, 2.8 Å with DFF40/DFF45 heterodimer, and 1.7 Å with FSP27 homodimer (Fig. 4a). Since the dimer form was dominant in the previous structural and biochemical study of the CIDE domain, we investigated how the helical filament was constructed by the DREP4 CIDE domain. In the helical filament structure of DREP4 CIDE, the different turns of the helix made no contact with each other. Due to the previous dimeric structure of the CIDE domain and the feature of the helical filament structure of DREP4 CIDE, we hypothesized that a 1:1 dimer of DREP4 CIDE is already stable, as a result of which, they do not need more contacts. To verify the hypothesis, we generated one mutant defective in the positively charged interface (K74E), and another defective in the negatively charged interface (D91K). Neither of K74E and D91K mutants produced complex peaks in size-exclusion chromatography, as expected (Fig. 4b). When the two mutants were mixed, incubated, and applied to size-exclusion chromatography, the peak corresponding to the dimer was produced, indicating that the two mutants interacted with each other using one positively charged interface of D91K mutant and another negatively charged interface of K74E mutant (Fig. 4b). Dimer formation due to charge interaction between two mutants was confirmed by MALS, performed in two different salt conditions, 150 mM and 1 M NaCl. Experimentally calculated molecular mass of the mixture of two mutants by SEC-MALS (performed in 150 mM and 1 M NaCl conditions) was 24 105 Da and 13 620 Da, respectively, indicating that the two mutants, D91K and K74E, formed dimer in solution, which was eventually disrupted under high salt concentration (Fig. 4c, d). The result showed that the basic construction unit of the helical filament structure of DREP4 CIDE is a dimer, formed by charge interaction (Fig. 4e).

### DREP4 CIDE specifically binds to histones H1 and H2

**Fig. 5:**
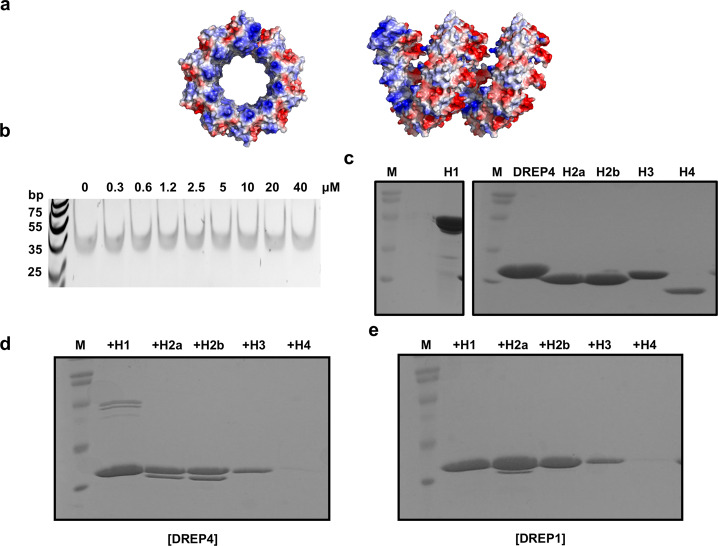
Specific in vitro interaction of DREP4 CIDE with histone. **a** Surface electrostatic figures of helical oligomer of DREP4 CIDE that showed charge distribution on the surface. Top view and side view are shown in the left panel and right panel, respectively. **b** DNA shift assay. The amounts of purified DREP4 CIDE added and mixed with DNA are indicated above the gel. **c** SDS-PAGE with purified protein samples that were used for in vitro pull-down assay. M indicates protein size marker and H indicates histone. **d**, **e** SDS-PAGE results of DREP4 CIDE (**d**) and DREP1 CIDE (**e**) pull-down assay with each histone subunit.

Based on the common feature of CIDE domains to be separable into two distinctly charged surfaces, the helical filament structure of DREP4 CIDE was considered to possess two differently charged surfaces, basic surface in the inner circular part and acidic surface in the outer circular part (Fig. 5a). Since the filament formation of apoptotic nuclease is critical for the rapid nucleosomal fragmentation of genomic DNA, DREP4 CIDE was initially considered to directly wrap up the chromosomal DNA by direct interaction with the latter. To analyze the direct interaction of DREP4 CIDE with DNA, we performed agarose gel-shift assay. As shown at Fig. 5b, DNA bands did not shift due to the addition of various concentrations of DREP4 CIDE protein, hence indicating that DREP4 CIDE did not interact directly with DNA.

Previous studies had indicated that histone H1 enhanced the nuclease activity of DFF40 [36, 37]. Since histone, especially H1, might be a candidate binding partner of the helical architecture of the CIDE domain of apoptotic nuclease, we performed histidine-tag pull-down assay with his-tagged CIDE proteins and untagged histone proteins, including H1, H2a, H2b, H3, and H4. All the histone proteins were prepared and loaded onto acrylamide gels for checking the band positions by SDS-PAGE (Fig. 5c and Supplementary Fig. 4a). Our pull-down analysis showed DREP4 CIDE to directly and specifically bind to histones H1, H2a, and H2b. Untagged histones H1, H2a, and H2b co-eluted with his-tagged DREP4 when the protein was mixed and loaded onto a Ni-NTA affinity column; however, histones H3 and H4 did not co-elute with DREP4 CIDE (Fig. 5d and Supplementary Fig. 4b). To check the specific interaction of DREP4 CIDE with histones, we performed the same pull-down analysis using DREP1 CIDE. Although a little trace of untagged histone H2a was detected, most histone failed to co-elute with DREP1 CIDE, indicating that histone interaction with DREP4 CIDE is highly specific (Fig. 5e and Supplementary Fig. 4c). Results of the present study supported the notion that histones H1, H2a, and H2b directly bind to DREP4 (DFF40), conferring DNA accessibility and stimulating nuclease activity of DREP4 (DFF40).

### E99K and I119C mutants of DREP4, with diminished enzymatic activity, failed to interact with histones

**Fig. 6:**
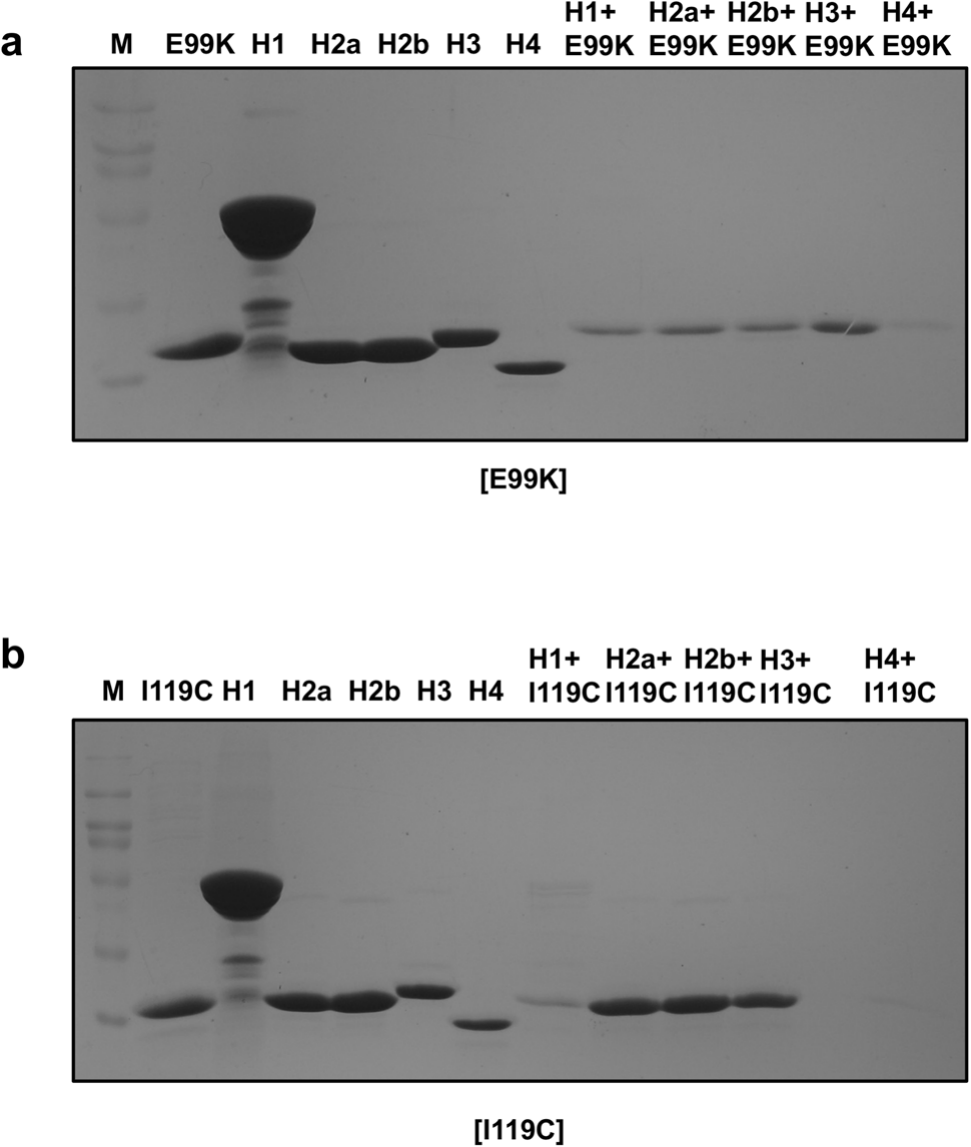
Histone binding-defective mutants DREP4, E99K, and I119C. SDS-PAGE results of E99K (**a**) and I119C (**b**) pull-down assay with histone subunits. The loaded protein samples are indicated above the corresponding lanes. M indicates protein size marker.

Among the mutants of DFF40 that can diminish the DNA fragmentation activity, W78C and D58K, which are not on the interface, remain enigmatic. Since these mutants might be related to the binding of histone, we tested the interaction of two mutants, E99K and I119C in DREP4 (corresponding to D58K and W78C in DFF40), with histones by pull-down study. According to the analysis, although most mutants still bound to histone subunits (Supplementary Fig. 5a–f), both E99K and I119C failed to interact with histones H1, H2a, and H2b (Fig. 6a, b). Based on structure analysis, we noticed E99K, which still possessed homo-oligomerization capacity but lost the histone-binding ability, to be located immediately adjacent to the homo-oligomeric interaction interface and exposed to the surface of the CIDE domain, whereas I119C, which showed the same pattern as E99K, was located immediately adjacent to the hinge loop and varied in the CIDE domain (Fig. 2f). I119 formed hydrophobic interaction with the neighboring I111, located in the CIDE domain (Fig. 2f, g). This study indicated that fixed hinge loop within the CIDE domain, which enables the movement of the linker loop and nuclease domain, might be critical for binding with histone and important for the proper functioning of the CIDE domain.

### Proposed model of the apoptotic DNA fragmentation process

**Fig. 7:**
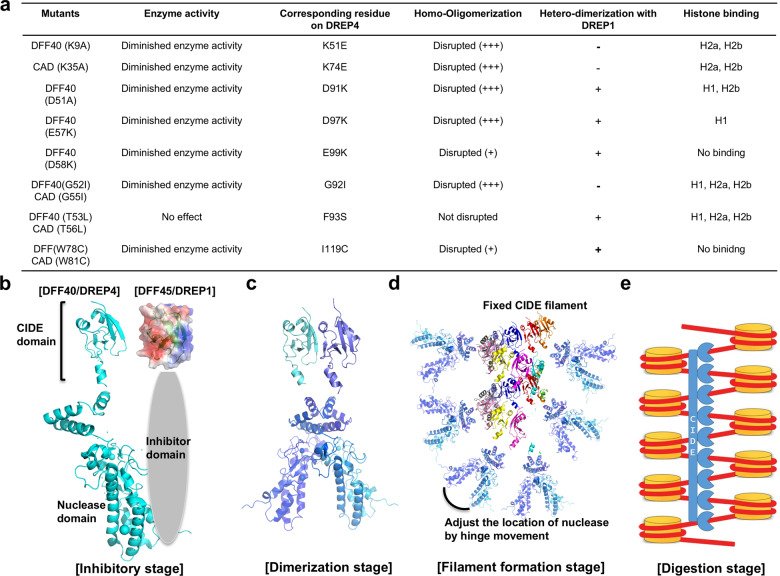
A model of apoptotic DNA fragmentation process at the molecular level. **a** Summarized table of previously and currently elucidated biochemical and enzymatic properties of DFF and its various mutants. **b**–**e** Proposed distinct stages of apoptotic DNA fragmentation: 1. inhibitory stage (**b**), 2. dimerization stage (**c**), 3. filament formation stage (**d**), and 4. digestion stage (**e**).

Based on our current studies, 1) showing the existence of hinge loop and linker helix that can adjust the location of the nuclease domain of DREP4 (DFF40), 2) revealing the homodimer of DREP4 (DFF40) as the basic filament building unit, and 3) indicating direct interaction of DFF40 CIDE with histone, along with the previous biochemical and structural studies, we proposed a model of the molecular process of apoptotic DNA fragmentation, which is a hallmark of apoptosis (Fig. 7a). In the absence of activation of apoptosis and caspases, DREP1 (DFF45) inhibits DFF40 (DREP4) via tight heteromeric interaction between CIDEs (Fig. 7b). The basic surface of DFF40 CIDE interacts with the acidic surface of DFF45 CIDE. Once the effector caspase is activated during apoptosis, it cleaves the inhibitor domain of DFF45 causing DFF45 to dissociate from DFF40. The released DFF40 initially forms a stable dimer, which is the basic functional unit of DFF40 (Fig. 7c). Soon after dimerization, DFF40 forms a helical filament-like oligomer, constructed by head-to-tail polymerization of DFF40 CIDE domain via charged interfaces (Fig. 7d). The helical oligomer of DFF40 wraps up chromatin via interaction between CIDE domain of DFF40 and histones H1, H2a, and H2b in the chromatin, leading to the cleavage of DNA with nucleosome unit, releasing approximately 180-bp DNA fragment (Fig. 7e). For the proper binding of DFF40 filament to histones in a specific site of chromatin, adjustment of the localization of nuclease domain via hinge loop and linker helix might be critical.

## Supplementary information

Supplemental figures

Checklist

## Data Availability

All data generated during this study are included in this published article and its supplementary information files.
