# Supplementary material for: Molecular basis of apoptotic DNA fragmentation by DFF40
Source: Cell Death Dis. 2022 Mar 2;13(3):198. doi: 10.1038/s41419-022-04662-7 (PMC8891305; doi:10.1038/s41419-022-04662-7)
Supplement: Supplementary file 1 — Supplemental figures [file 41419_2022_4662_MOESM1_ESM.docx]

**Molecular basis of apoptotic DNA fragmentation by DFF40**

Hyun Ji Ha^1^ and Hyun Ho Park^1,2*^

^1^College of Pharmacy, Chung-Ang University, Seoul 06974, Republic of Korea

^2^Department of Global Innovative Drugs, Graduate School of Chung-Ang University, Seoul 06974, Republic of Korea

^*^Hyun Ho Park, Phone: +82-2-820-5930. Fax: +82-53-810-4516. E-mail: [xrayleox@cau.ac.kr](mailto:xrayleox@cau.ac.kr)

**Supplementary information**

**
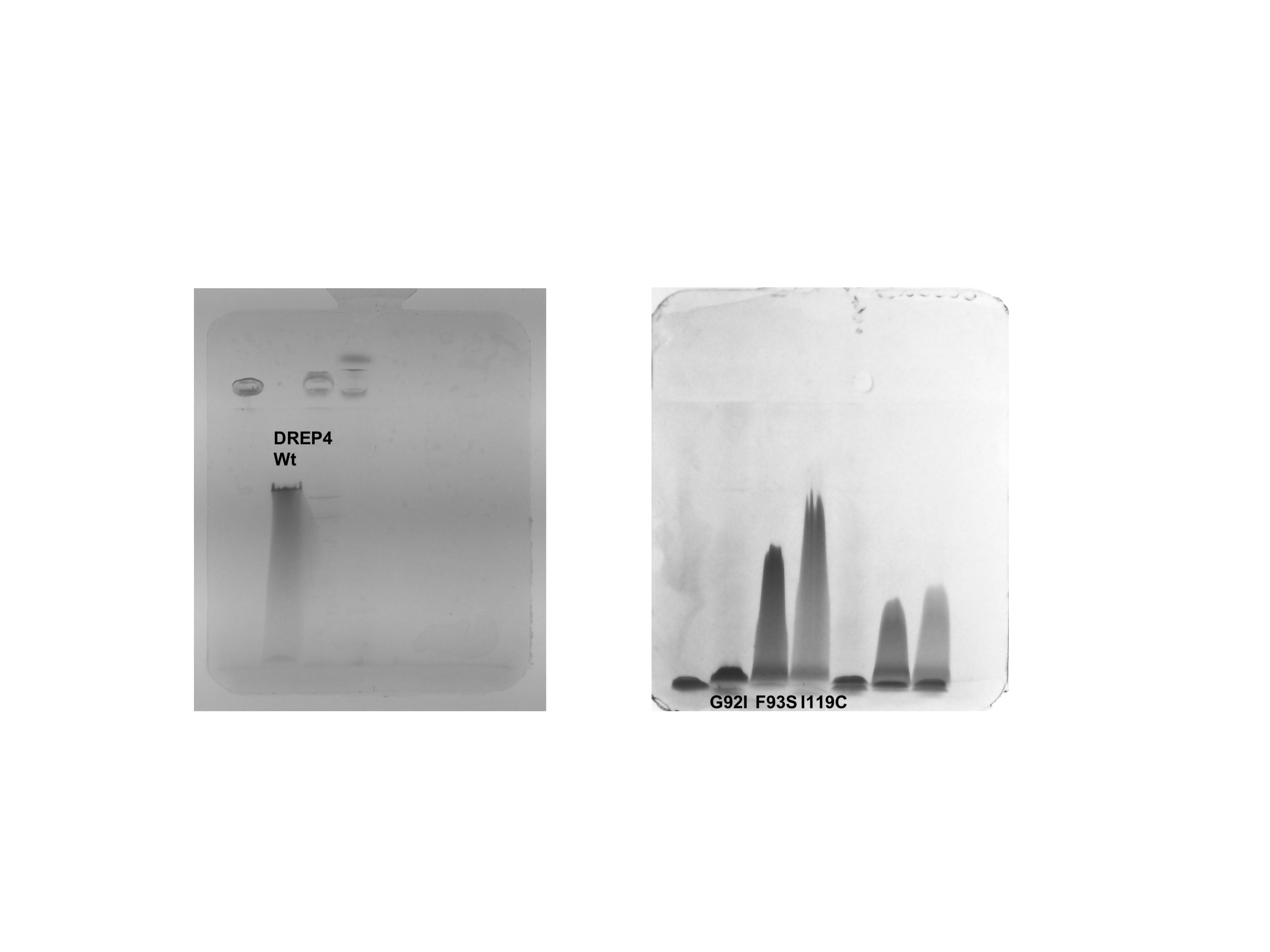
**

**Fig. S1.** Uncropped gels used for generating figure 2e. Cropped and used for the figure generation was labelled.

**
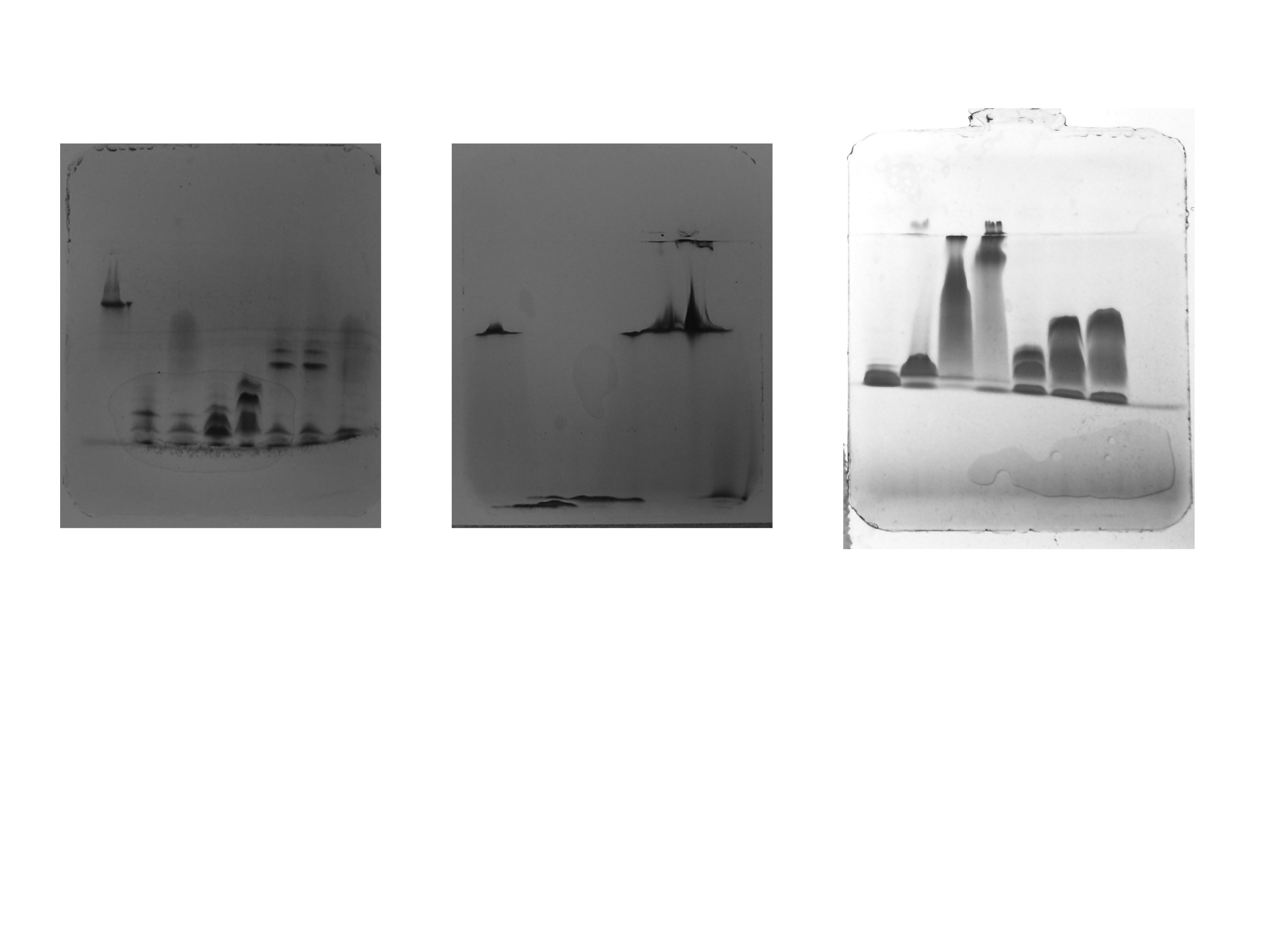
**

**Fig. S2.** Uncropped gels used for generating figure 3.

**
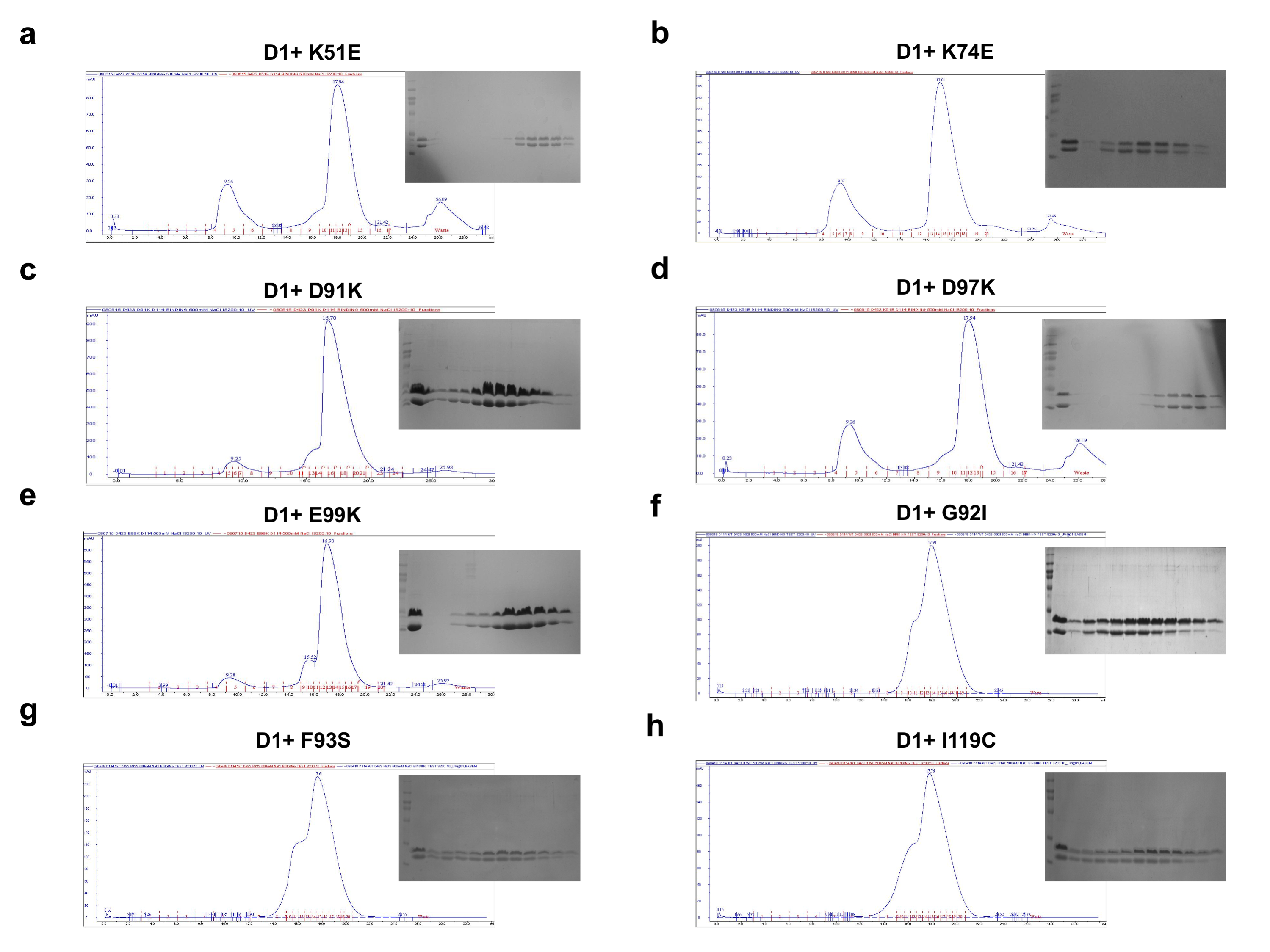
**

**Fig. S3.** Interaction analysis of various DREP4 mutants with DREP1. (a~h) Profiles of size-exclusion chromatography with SDS-PAGE performed with samples from eluted peak. D1 indicates DREP1 CIDE. Each mutant of DREP4 CIDE, K51E (a), K74E (b), D91K (c), D97K (d), E99K (e), G92I (f), F93S (g), and I119C (h), were mixed with DREP1 CIDE for 1 hour before applying to size-exclusion chromatography.

**
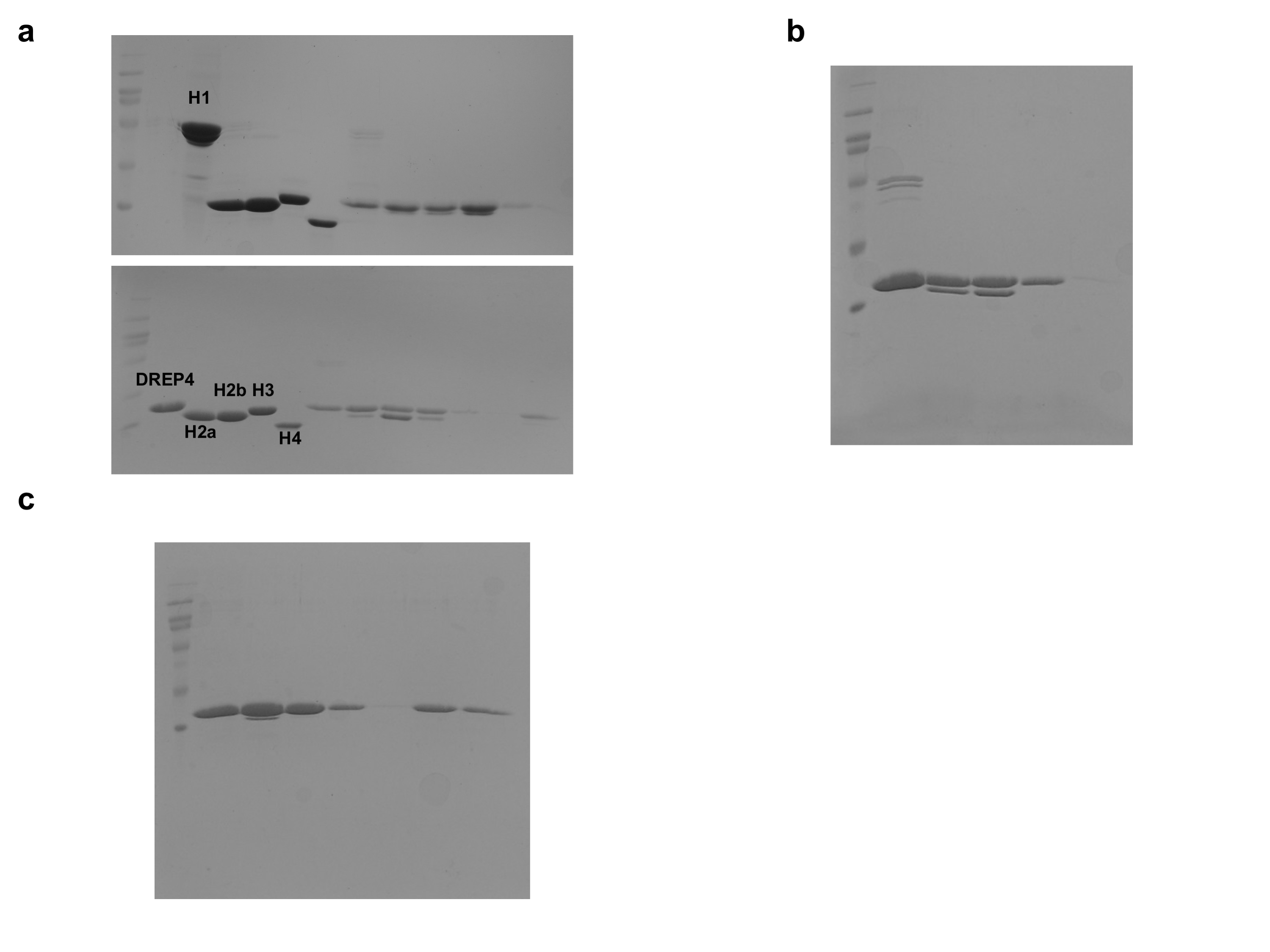
**

**Fig. S4.** Uncropped gels used for generating figure 5. a. Uncropped gels used for generating figure 5c. Cropped and used for the figure generation was labelled. b. Uncropped gel used for generating figure 5d. c. Uncropped gel used for generating figure 5e.

**
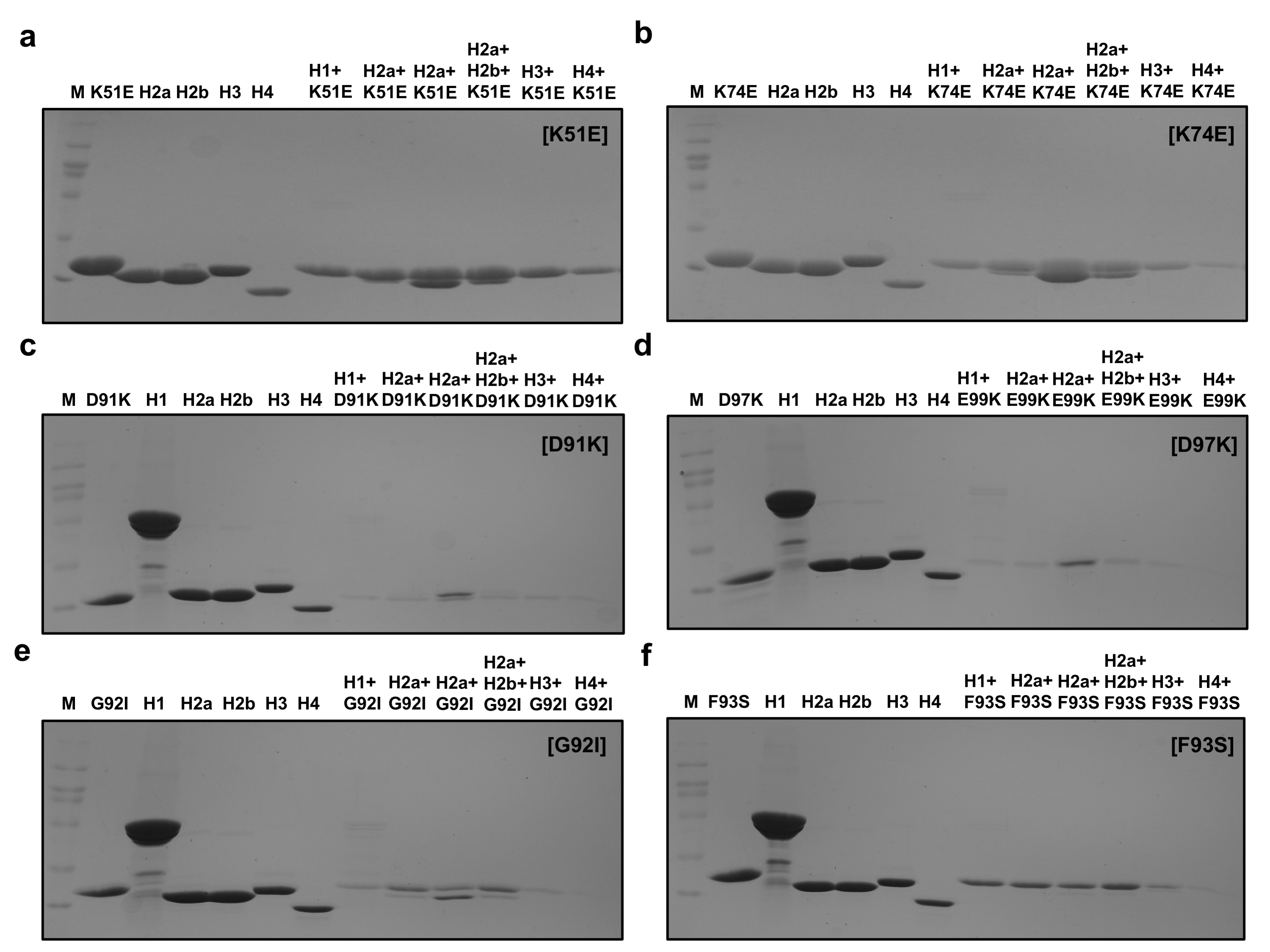
**

**Fig. S5.** Binding analysis of various mutants of DREP4 with histone subunits. SDS-PAGEs showing each mutant pull-down assay, K51E (a), K74E (b), D91K (c), D97K (d), G92I (e), and F93S (f) with histone subunits. The loaded protein samples were indicated above the corresponding lanes. M indicated protein size marker.
